# A *Trypanosoma brucei* Protein Required for Maintenance of the Flagellum Attachment Zone and Flagellar Pocket ER Domains

**DOI:** 10.1016/j.protis.2011.10.010

**Published:** 2012-07

**Authors:** Sylvain Lacomble, Sue Vaughan, Michaël Deghelt, Flávia Fernandes Moreira-Leite, Keith Gull

**Affiliations:** a Sir William Dunn School of Pathology, University of Oxford, Oxford OX1 3RE, United Kingdom; b Oxford Brookes University, Headington Hill site, Headington, Oxford OX3 0BP, United Kingdom

**Keywords:** ERES, ER exit sites, FAZ, flagellum attachment zone, FAZ ER, flagellum attachment zone-associated endoplasmic reticulum, MSP, major sperm protein, MtQ, microtubule quartet, PFR, paraflagellar rod, VAP, VAMP-associated protein, Cellular electron tomography, endoplasmic reticulum, flagellar proteome, flagellum attachment zone, *Trypanosoma brucei*, VAMP-associated protein.

## Abstract

Trypanosomes and Leishmanias are important human parasites whose cellular architecture is centred on the single flagellum. In trypanosomes, this flagellum is attached to the cell along a complex flagellum attachment zone (FAZ), comprising flagellar and cytoplasmic components, the integrity of which is required for correct cell morphogenesis and division. The cytoplasmic FAZ cytoskeleton is conspicuously associated with a sheet of endoplasmic reticulum termed the ‘FAZ ER’. In the present work, 3D electron tomography of bloodstream form trypanosomes was used to clarify the nature of the FAZ ER. We also identified TbVAP, a *T. brucei* protein whose knockdown by RNAi in procyclic form cells leads to a dramatic reduction in the FAZ ER, and in the ER associated with the flagellar pocket. TbVAP is an orthologue of VAMP-associated proteins (VAPs), integral ER membrane proteins whose mutation in humans has been linked to familial motor neuron disease. The localisation of tagged TbVAP and the phenotype of TbVAP RNAi in procyclic form trypanosomes are consistent with a function for TbVAP in the maintenance of sub-populations of the ER associated with the FAZ and the flagellar pocket. Nevertheless, depletion of TbVAP did not affect cell viability or cell cycle progression.

## Introduction

The single flagellum of *T. brucei* has important functions in the life cycle as well as in the cell cycle of the parasite. It is used for cell motility and for attachment to the salivary gland of the insect vector, during differentiation to the infective form (Tetley and Vickerman 1985). Knockdown by RNAi of a variety of flagellar components is lethal to mammalian bloodstream form trypanosomes (Alsford et al. 2011; Broadhead et al. 2006). Also, we have shown that in vivo knockdown of a flagellar protein required for cell motility is capable of clearing *T. brucei* parasitemia in mice (Griffiths et al. 2007). Although relatively mild cell motility defects might be tolerated in the bloodstream form (Ralston et al. 2011), the majority of data available to date support an essential role for a wide variety of protein types with flagellar functions in the bloodstream form of the parasite.

Importantly, the intricate cytoarchitecture of the trypanosome cell cytoskeleton is defined by the flagellum polarity and position, and cells without a flagellum are defective in cell morphogenesis (Davidge et al. 2006; Kohl et al. 2003). At its proximal end, the flagellum is physically linked to the kinetoplast (the mitochondrial DNA) (Ogbadoyi et al. 2003; Robinson and Gull 1991) and is associated with a complex set of cytoskeletal and membrane components in the flagellar pocket area (Lacomble et al. 2009a). During cell division, new flagellum formation orchestrates the coordinated morphogenesis and segregation of membrane and cytoskeletal elements associated with the proximal end of the flagellum (Absalon et al. 2008; Lacomble et al. 2010; Robinson and Gull 1991).

The flagellum is also indirectly linked to the cytoskeleton along its length via its attachment to the cell body at the flagellum attachment zone, or FAZ (Vaughan et al. 2008; Vickerman 1969). The *T. brucei* flagellum emerges from the flagellar pocket - an invagination of the plasma membrane at the posterior of the cell – and is attached to the cell body along the FAZ, which runs from posterior to anterior. Analyses of cells defective in flagellum or FAZ formation suggest that flagellum attachment to the cell at the FAZ has a major role in the coordination of cell morphogenesis in trypanosomes (Kohl et al. 2003; Vaughan 2010; Vaughan et al. 2008). Morphologically, the FAZ is defined by components on both the flagellum and the cytoplasmic side of the region of adhesion between the flagellar membrane and the plasma membrane (Lacomble et al. 2009a). On the flagellar side, filamentous structures link the flagellar membrane to the paraflagellar rod (PFR) (Portman and Gull 2010), a paracrystalline lattice like component of the flagellar cytoskeleton that runs in parallel with the canonical 9 + 2 microtubular axoneme. On the cytoplasmic side of the FAZ, a cytoskeletal structure termed the FAZ filament is present below the cell membrane, immediately opposite to the point where the flagellar membrane is linked to the PFR (Kohl et al. 1999). To the left of the FAZ filament, when the cell is viewed from the posterior, lies a group of 4 specialised microtubules (Taylor and Godfrey 1969; Vickerman 1969). This ‘microtubule quartet’ (MtQ) originates at the proximal end of the flagellum (the one closest to the basal bodies) and runs towards the anterior of the cell alongside the FAZ filament (Lacomble et al. 2009a). These are the only microtubules associated with the flagellar pocket membrane, and they are anti-parallel to the remaining cortical microtubules, having their plus ends at the anterior of the cell (Robinson et al. 1995). Along the FAZ, the microtubules of the MtQ are closely associated with a sheet of ER, being interdigitated by ER membranes. This FAZ-associated ER will herein be referred to as FAZ ER. The relationship between this ‘cortical’ ER domain and the central ER has not been described in detail, although connections have been observed between the FAZ ER and the central ER (Vickerman 1969) or the outer nuclear membrane (Taylor and Godfrey 1969). It is possible that maintenance of ER morphology and physiology relies on the intimate association between the ER and the FAZ cytoskeleton. In this context, it is interesting to note that the *T. brucei* ERES (for ER exit sites), an important domain of the ER, has been localized in close proximity to the FAZ (Sevova and Bangs 2009).

We have performed a 3D reconstruction of the FAZ ER at different positions along the long axis of the cell, in bloodstream form trypanosomes. This analysis provided a detailed view of the association between the FAZ ER and the central ER, and showed that ribosomes decorate the membrane of the FAZ ER, clarifying the nature of this ER domain. Interestingly we have identified in our flagellar proteome (Broadhead et al. 2006) a trans-membrane protein required to maintain the FAZ ER and also the ER associated with the flagellar pocket membrane. We named this novel protein TbVAP, due to its homology with VAMP associated proteins (VAPs), type II integral ER membrane proteins found in a diverse range of eukaryotes (Lev et al. 2008). In humans, a single missense mutation in the VAP-B gene has been linked to three forms of familial motor neuron disease (Nishimura et al. 2004, 2005). The localisation of tagged TbVAP suggests that this protein is present in the many different ER domains of a trypanosome cell, including the FAZ ER and the flagellar pocket associated ER. Cells depleted of TbVAP showed considerably less ER associated with the FAZ, and also with the flagellar pocket membrane. Despite the striking effect of TbVAP knockdown in the architecture of specific ER domains, but consistent with suppression of VAP function in other micro-organisms (Kagiwada et al. 1998; Loewen et al. 2007), TbVAP RNAi did not affect cell viability or cell cycle progression in culture conditions.

## Results

### The FAZ Microtubule Quartet (MtQ) and the ER

Despite the importance of the flagellum/FAZ area for *T. brucei* cell morphogenesis, little attention has been given to the FAZ ER beyond its original descriptions in 1969 (Taylor and Godfrey 1969; Vickerman 1969). The conspicuous morphology of the FAZ ER as observed in cross-sections of transmission electron microscopy (TEM) shows a sheet of endoplasmic reticulum typically associated with 3 to 4 microtubules of the MtQ at the FAZ. It has been observed that the FAZ ER connects with central ER membranes (Vickerman 1969) and with the outer nuclear membrane (Taylor and Godfrey 1969), but no detailed morphological analysis of these links has been performed, and it is unclear whether they are found throughout the FAZ or only in one or a few specific positions (for example, only close to the nucleus). Another important issue that needs clarification is whether the FAZ ER is rough or smooth. It was originally described by K. Vickerman as a “diverticulum of the granular reticulum” (Vickerman 1969), implying that it derived from the rough ER. However, it is generally described in the literature as smooth ER, possibly because the association of ribosomes with the FAZ ER is not always clear, or simply due to the lack of a careful morphological analysis of this ER domain.

In order to clarify the morphology and nature of the FAZ ER, we performed 3D reconstructions of the FAZ ER by electron microscopy tomography in the bloodstream form of *T. brucei* (Fig. 1, and Supplementary Material Movies S1a, S1b, S2a and S2b). We observed connections between the FAZ ER and the central ER at different positions along the length of the FAZ. Data from two representative sections along the major axis of the cell are shown here. The section in Figure 1 (B and C), and movies S1a and S1b corresponds to an area immediately after the flagellum emerges from the flagellar pocket, identified by the presence of the Golgi complex. The section shown in Figure 1 (D and E) and movies S2a and S2b is from a different area along the major axis of the cell, most likely between the nucleus and the anterior end, since it shows the FAZ but does not include either the Golgi or the nucleus.

In any section of the FAZ, connections were observed between the FAZ ER and the central ER (arrows in Fig. 1B, C and E, and Supplementary Material Movies S1a, S1b, S2a and S2b). These connections were not long sheets of ER, but short ‘tube-like’ bridges which could be reconstructed in their entirety from 150-300 nm TEM cross sections of cells (Fig. 1, C and E). The tube-like morphology of these bridges is the likely reason why they are often missed in sections through the FAZ, despite the fact that they appear to be found along the FAZ, and not only near the nucleus. Although our analysis was performed in the bloodstream form of *T. brucei*, examination of our vast collection of micrographs from procyclic trypanosomes showed that bridges are often found between the FAZ ER and the central ER in this life cycle form as well as in bloodstreams. Our tomography data clearly show ribosomes in association with the FAZ ER (Fig. 1, D and E, and Supplementary Material Movies S2a and S2b), confirming the earlier description that this is a domain of rough ER, rather than merely smooth ER.

As mentioned above, the FAZ ER is usually found closely associated with 3 or 4 microtubules of the MtQ. Whenever it is found associated with only 3 of the 4 MtQ microtubules, a circular structure described as a “reduced microtubule” (Vickerman 1969) is found immediately to the right of the MtQ (Fig. 1F and G). Upon 3D reconstruction, this so called “reduced microtubule” (arrowhead in Fig. 1E) appears as a thin tube running parallel to the MtQ. We have observed this structure carefully in TEM sections and we believe that it is not a microtubule, but a thin membrane tubule (Fig. 1F and G). It is possible that this structure is part of the FAZ ER, which often occupies the same region of the cell (for example, as in Fig. 1A and B). However, during 3D reconstruction, we did not observe links between the “reduced microtubule” and the FAZ ER (Fig. 1E and movie S2a). Nevertheless, the walls of this thin tubule are clearly of a membranous nature indistinguishable from that of the neighbouring FAZ ER (Fig. 1F). Also, we have noted that the rightmost region of the FAZ ER (the one closest to the FAZ filament) is somewhat more ‘plastic’ and, in some stretches along the FAZ, appears detached from the remainder of the structure (Supplementary Material Movies S1a and S1b). In light of this, it is possible that the reduced microtubule is, in fact, an alternative ‘configuration’ of the rightmost region of the FAZ ER, found in some areas along the FAZ.

### A *T. brucei* VAMP-associated Protein (VAP) Orthologue

The FAZ is comprised of a complex network of interactions between cytoskeletal and membrane components (Sherwin and Gull 1989). The only known membrane component that participates in this network is Fla1, a trans-membrane protein required for attachment of the flagellum to the cell body (LaCount et al. 2002; Nozaki et al. 1996). In order to identify other membrane proteins linked to the FAZ cytoskeleton, we searched our flagellar proteome (Broadhead et al. 2006) for proteins containing a predicted trans-membrane domain (TMD). It is important to note that this proteome was produced by analysing a 1 M NaCl-insoluble fraction of detergent extracted cell. Therefore, the only membrane proteins expected to be part of the *T. brucei* flagellar proteome are those stably linked to the flagellum/FAZ cytoskeleton.

Reassuringly, Fla1 was amongst the 9 membrane proteins identified in the *T. brucei* flagellar proteome. Amongst the 8 remaining putative trans-membrane proteins, the product of gene Tb11.01.4810 was particularly interesting. This gene encoded a putative protein of 216 amino-acids and predicted molecular weight of 23.9 kDa containing a major sperm protein (MSP) domain at the N-terminus and a TMD at the C-terminus (Fig. 2A). MSP is a filament forming protein that mediates amoeboid motility in nematode sperm (Roberts and Stewart 1995). Bioinformatic comparison with 50 other proteins with an N-terminal MSP domain and a C-terminal TMD revealed that the product of locus Tb11.01.4810 encoded a *T. brucei* orthologue of VAMP-associated proteins (VAPs) (Fig. 3B). Therefore, we renamed this protein TbVAP.

VAPs are type II integral ER membrane proteins that have been implicated in the regulation of a plethora of important cellular processes, including membrane trafficking (Nishimura et al. 1999; Skehel et al. 1995), lipid transport and metabolism (Kagiwada and Zen 2003; Kawano et al. 2006; Nikawa et al. 1995), the unfolded protein response (Brickner and Walter 2004; Kanekura et al. 2006) and microtubule organization (Pennetta et al. 2002; Skehel et al. 2000). Within the N-terminal MSP domain, VAPs have a well conserved signature motif which is found in TbVAP (Fig. 2B), including a proline residue whose mutation to a serine in human VAP-B is linked to three forms of familial motor neuron disease (Nishimura et al. 2004, 2005). As shown in Figure 2B, a VAP signature motif is found within the MSP domain of TbVAP, between amino-acids 41 and 58, and the proline residue found mutated in familial motor neuron diseases is conserved in TbVAP. However, TbVAP lacks a C-terminal GXXXG dimerization motif, which is also missing in many other VAPs (Lev et al. 2008).

We have identified orthologues of TbVAP in the genomes of *T. b. gambiense*, *T. congolense*, *T. cruzi*, and 3 species of *Leishmania* (Supplementary Material Fig. S1). Also, the *T. vivax* genome database contains a partial sequence of a possible TbVAP orthologue. All sequences have a predicted C-terminal trans-membrane domain. The trypanosome TbVAP orthologues have a conserved VAP motif, while the *Leishmania* proteins contain a very degenerate VAP motif which lacks the important proline residue mutated in familial motor neuron disease.

### A Pool of TbVAP is Stably Linked to Cytoskeleton at the FAZ

Given that TbVAP is a putative integral ER membrane protein present in the flagellum proteome, we hypothesized that it could be involved in linking the ER to the MtQ at the FAZ. In order to test this hypothesis and to investigate the importance of the ER association with the FAZ, we decided to establish the localisation and function of TbVAP.

For localisation studies, one of the two endogenous copies of TbVAP in the genome was tagged with an N-terminal Ty epitope tag (Bastin et al. 1996; Kelly et al. 2007). Procyclic trypanosome cells expressing Ty-TbVAP were fixed and analysed by immunofluorescence using the BB2 antibody, which recognizes the Ty epitope tag.

In Ty-TbVAP expressing cells fixed with paraformaldehyde, strong BB2 labelling was observed along the FAZ and at the periphery of the flagellar pocket (Fig. 3A). BB2 signal was also observed in reticulate structures throughout the cytoplasm of paraformaldehyde fixed cells, consistent with an ER localisation. In Ty-TbVAP expressing cells fixed in methanol (Fig. 3B), the FAZ was clearly labelled with BB2, and labelling was also observed in the flagellar pocket area, but no labelling was seen in other cytoplasmic structures. Finally, when detergent treated cells were labelled with BB2 (Fig. 3C), a clear signal was observed only along the FAZ. Taken together, these results show that a proportion of TbVAP (approx. 60% of the total TbVAP in the cell, Supplementary Material Fig. S2) is stably linked to the cytoskeleton at the FAZ and, therefore, resistant to detergent (or methanol) extraction prior to fixation.

To refine our localisation of the cytoskeletal-associated pool of Ty-TbVAP within the FAZ, we double labelled Ty-TbVAP expressing cells with BB2 and the monoclonal antibody L6B3, which recognises the FAZ filament component FAZ1 (Fig. 3D) (Kohl et al. 1999). Double labelling of methanol fixed cells with BB2 and L6B3 showed co-localisation of FAZ1 and Ty-TbVAP along the FAZ, although subtle differences could be observed between BB2 and L6B3 lablelling at the posterior and anterior extremities of the FAZ (Fig. 3D). While BB2 labelling was strong at the anterior of the FAZ (arrowheads in Fig. 3D), L6B3 labelling was weaker in this area. In contrast, close to the flagellar pocket (arrows in Fig. 3D), a short stretch of the FAZ appeared devoid of Ty-TbVAP, but contained FAZ1.

### TbVAP RNAi Severely Decreases the FAZ ER

To investigate the function of TbVAP, we produced a construct for doxycycline-inducible RNA interference (RNAi) of TbVAP based on the p2T7-177-GTW vector (Lacomble et al. 2009b). This construct was transfected in procyclic form trypanosomes. After transfection, selection and cloning, we confirmed that cells were capable of doxycycline-inducible knockdown of Ty-TbVAP protein expression by western blotting (Fig. 4A). We chose to analyse the phenotype of TbVAP RNAi after 72 h of induction, when no Ty-TbVAP protein could be detected by western blotting (Fig. 4A).

To investigate the possibility that TbVAP facilitates the association of the ER with the FAZ cytoskeleton, we compared the FAZ region of induced and non-induced cells as seen by thin-section transmission electron microscopy (TEM) (Fig. 4B-F). Sections in the region of the flagellum of non-induced cells showed the typical FAZ morphology (Fig. 4C). Inside the flagellum, filaments linked the PFR to the flagellar membrane, immediately opposite to the location of the FAZ filament in the cytoplasm. The MtQ with associated ER was observed to the left of the FAZ filament, when the cell was viewed from the posterior. TbVAP RNAi had a major negative impact on the ER associated with the MtQ (Fig. 4B-F). There was ER associated with 3 or 4 microtubules of the MtQ in less than 10% of transverse TEM sections of the FAZ after 72 h of induction of TbVAP RNAi (n = 64), compared to 73% of sections of the FAZ of non-induced cells (n = 46) (Fig. 4B). In approximately 50% of sections across the FAZ of induced cells, no microtubule of the MtQ had associated ER (Fig. 4B and D), with approximately 40% of the remaining sections showing a single microtubule of the MtQ (more often the one closest to the FAZ filament) associated with an ER membrane profile (Fig. 4B and E). Statistical analysis of this dataset showed a highly significant difference between induced and non-induced samples regarding the extent of the ER associated with the MtQ (Chi-square test, P < 0.01).

As mentioned previously, we detected TbVAP at the periphery of the flagellar pocket of procyclic *T. brucei* by immunofluorescence (Fig. 3A). This could represent TbVAP in the ER subtending the flagellar pocket membrane (Fig. 4H). Given the importance of the flagellar pocket as the sole site of endo- and exocytosis in the cell, we decided to investigate the impact of TbVAP RNAi on the flagellar pocket associated ER. Thin-section TEM images of the flagellar pocket from non-induced and induced TbVAP RNAi cells (n = 74 and 76, respectively) were compared regarding the extent of ER association with the flagellar pocket. For each section across the flagellar pocket, the length of ER and flagellar pocket membranes were measured using the IMOD package (Kremer et al. 1996) and the percentage of the length of the sectioned flagellar pocket membrane with associated ER was calculated (Fig. 4G). There was significantly less ER associated with the flagellar pocket membrane of cells after 72 h of induction of TbVAP RNAi (Fig. 4G-I). We also observed a small but statistically significant decrease in the perimeter of the flagellar pocket in induced samples compared to non-induced (2.7 ± 0.2 μm and 2.9 ± 0.3 μm, respectively), suggesting that knockdown of TbVAP results in a small decrease in the size of the flagellar pocket.

### In vitro Growth is not Compromised by TbVAP RNAi

Despite the impact of TbVAP RNAi on the architecture of the ER at the FAZ and at the flagellar pocket, cells where TbVAP RNAi was induced remained viable. Population growth analysis revealed no difference between induced and non-induced cells in regular culture conditions, throughout a period of 72 h of induction (Fig. 5A). DAPI-stained cells from an asynchronously dividing population can be placed into one of three stages of the cell cycle: 1) 1K1N cells, where neither the kinetoplast (K) nor the nucleus (N) have divided (corresponding to the G1/S stage of the cell cycle); 2) 2K1N cells, where kinetoplast (but not nuclear) division has occurred (G2/M phase cells); and 2K2N cells, where both the kinetoplast and the nucleus have divided, prior to cytokinesis (Sherwin and Gull 1989; Woodward and Gull 1990). Induction of TbVAP RNAi did not affect the proportion of cell cycle stages (Fig. 5B).

## Discussion

In this work we have provided a detailed 3D view of the trypanosome FAZ ER and have described a novel trypanosome protein, TbVAP, required for maintenance of this conspicuous ER domain. EM tomography reconstruction of the FAZ ER showed that it is clearly linked to the central ER via tube-like bridges found along the FAZ. This firmly establishes that the ER network in trypanosomes is anchored to the cytoskeleton at the FAZ, via the FAZ ER. We have also examined closely the nature of the FAZ ER, determining that it is a domain of rough, rather than smooth, ER, which confirms an earlier description by Keith Vickerman (1969).

In TEM cross-sections through the FAZ of wild type trypanosomes, two alternative FAZ ER morphologies have been observed: 1) the FAZ ER is closely associated to and interdigitates all 4 microtubules of the MtQ; or 2) the FAZ ER is closely associated to and interdigitates only 3 microtubules of the MtQ, while a “reduced microtubule” (Vickerman 1969) is found immediately to the right of the MtQ, when the cell is viewed form the posterior. Upon careful observation and 3D reconstruction of the so-called “reduced microtubule”, we believe that this structure is not a microtubule but a thin membrane tubule that runs parallel to the MtQ. We have not observed a consistent rationale for the slight variation of ER association with the MtQ. These images may represent different views of a dynamic process. However, given our studies it is wise to refer to this structure when seen as the FAZ tubule rather than a reduced microtubule.

We have shown that the normal morphology of the FAZ ER as described above is drastically changed upon induction of RNAi for TbVAP, the *T. brucei* orthologue of VAMP-associated proteins. Most TEM cross-sections through the FAZ of induced TbVAP RNAi cells showed little or no FAZ ER in association with the MtQ. The most well established function of VAPs is the regulation of lipid transport and metabolism, although these proteins have also been implicated in a diverse range of cellular processes including the regulation of membrane trafficking, the unfolded protein response, and linking the ER to the microtubule cytoskeleton (Lev et al. 2008). In light of the proposed functions of VAPs, and given the phenotype of TbVAP RNAi, we hypothesise that this protein has a major role in the formation and/or maintenance of the FAZ ER by one or both of the following mechanism: 1) linking ER membranes to the MtQ microtubules (directly or indirectly); or 2) regulating the dynamic interchange of vesicles between the FAZ ER and the central ER. Aside from its role in FAZ ER maintenance, our results also show that TbVAP is important for the maintenance of at least one other domain of the ER: the flagellar pocket associated ER. The fact that TbVAP RNAi does not reduce population growth argues against the possibility that this protein has a major function in maintenance of the ER as a whole. Also, immunofluorescence data provided no clear indication of a reduction in the central ER of the cell upon RNAi of TbVAP (not shown).

Interestingly, the sequence conservation of TbVAP orthologues across the Trypanosomatida supports our hypothesis that TbVAP plays an important role in the maintenance of the FAZ ER. *T. brucei* TbVAP and its orthologues in trypanosome species have a well conserved VAP signature motif, aside from extensive overall sequence similarity, in agreement with a function in the maintenance of the FAZ ER, which has been described in the American trypanosome *T. cruzi* (Rocha et al. 2006) as well as in different species of African trypanosomes (Taylor and Godfrey 1969; Vickerman 1969). Conversely, the *Leishmania* TbVAP orthologues have a very divergent VAP motif and may not function as VAPs. Indeed, *Leishmania* may not require FAZ ER maintenance, since they lack an extensive, linear flagellum attachment zone.

Mouse, Human and Drosophila VAPs have been shown to associate with microtubules in vivo or in vitro (Pennetta et al. 2002; Prosser et al. 2008; Skehel et al. 2000), and co-precipitation of human VAPA and VAPB with microtubules depends on the MSP domain (Prosser et al. 2008). It has been proposed that complexes containing VAPs physically link ER membrane to the microtubule network, providing anchor points for ER expansion driven by motor proteins, and forming stable ER membrane sites for protein folding and assembly (Amarilio et al. 2005; Prosser et al. 2008). In a similar manner, anchoring of the trypanosome ER network to the microtubule cytoskeleton by TbVAP could help in the establishment and maintenance of correct ER architecture and function. However, cell viability and cell cycle progression are not compromised when the FAZ ER and the flagellar pocket associated ER are severely reduced by TbVAP RNAi. It remains possible that complete ablation of these ER domains would compromise cell viability, while the remnants of these structures found in cells depleted of TbVAP are sufficient to maintain essential ER architecture and function, safeguarding cell viability.

Incomplete disappearance of the FAZ and flagellar pocket associated ER domains after TbVAP RNAi could be due to the presence of residual TbVAP in induced cells, undetectable by western blotting with the BB2 antibody. Alternatively, one can speculate that initiation and a level of maintenance of these ER domains to their full extent might involve a TbVAP-independent pathway, not affected by TbVAP RNAi. Combined ablation of TbVAP and proteins from the TbVAP-independent pathway might be required to compromise the FAZ and flagellar pocket ER domains to an extent that will affect cell viability. This was shown for the yeast VAP homologue Scs2, deletion of which reduces the cortical ER in yeast, while cells remain viable (Loewen et al. 2007). Combined deletion of Scs2 and other non-essential genes implicated in cortical ER maintenance further reduces the cortical ER and leads to a severe growth defect (Loewen et al. 2007). It is important to note that, while trypanosomes depleted of TbVAP are viable in vitro, TbVAP function might be required in vivo.

It seems unlikely that TbVAP functions in lipid metabolism or in the UPR response, an adaptive response to the accumulation of improperly folded proteins in the ER. Trypanosomes appear to lack a conventional UPR response (Izquierdo et al. 2009; Koumandou et al. 2008), although there is evidence for quality control monitoring of protein folding in the ER (Field et al. 2010). Knockdown of proteins believed to function in this quality control system produces slow growth and gross morphological defects (Field et al. 2010) not observed upon TbVAP RNAi. In the case of lipid metabolism, knockout of yeast Scs2p leads to a rise in the concentration of phosphatidylcholine, which inhibits a protein required for inositol biosynthesis (INO1), thereby causing inositol auxotrophy (Kagiwada and Zen 2003). In trypanosomes, increases in the level of phosphatidylcholine resulted in a cytokinesis defects (Signorell et al. 2008). The fact that no cytokinesis defect is associated with TbVAP knockdown argues against the hypothesis that TbVAP is involved in lipid metabolism in a similar manner to its yeast counterpart.

The identification of proteins involved in orchestrating the sub-pellicular arrangement of filaments and microtubules is increasing apace. The ER associated with the MtQ at the FAZ is a classical feature of the trypanosome cellular organisation. This study documenting TbVAP as an important player in this association provides insight to a likely protein cohort responsible for the complexity of these interactions and functions. The MtQ provides an antiparallel ‘seam’ within the sub-pellicular array. It is an intriguing question as to why there is this intimate association of ER with only these 4 microtubules. One is tempted to speculate that the association is dynamic and directional in terms of ER movement. Membrane could be fed onto the MtQ from the pocket area or membrane delivered in a coordinated way to the pocket area for the exocytotic and endocytotic processes. For instance the huge amount of VSG/Procyclin to be synthesised and presented to the surface may necessitate this type of directional factory. Development of temporal imaging technologies applied to residence marker proteins in this ER may provide support for these speculations of an intensive, directional membrane flow.

## Methods

**Cell culture:** *Trypanosoma brucei brucei* procyclic forms (strain 427) were cultured at 28 °C in SDM 79 medium supplemented with 10% v/v foetal calf serum. Bloodstream form cells were maintained at 37 °C in 5% CO_2_ and HMI-9 medium containing 10% fetal bovine serum and 10% Serum Plus (JRH Biosciences). For induction of TbVAP RNAi in stably transfected strains doxycycline was added to the culture medium to a final concentration of 1 μg ml^−1^.

**Electron microscopy and 3D tomography:** Conventional transmission electron microscopy (TEM) and electron microscopy tomography of trypanosomes were performed as previously described (Lacomble et al. 2009a).

**Bioinformatics:** Using the online tool TMHMM2 (Kall et al. 2004), we identified the product of gene Tb11.01.4810 as a predicted trans-membrane protein in the *Trypanosoma brucei* flagellar proteome (Broadhead et al. 2006). An iterative Hidden Markov Model of the Tb11.01.4810 protein was built and used to search for orthologues in 36 different eukaryote genomes (with an e-value cutoff of 10^−10^). The 168 possible orthologues identified were then screened using TMHMM2 and an in-house pfam (Finn et al. 2008) Hidden Markov Model in order to identify proteins with a similar domain structure to that of Tb11.01.4810 (i.e., having an N-terminal MSP domain and a single C-terminal trans-membrane domain). This screen identified 47 unique protein sequences, which were aligned with the Tb11.01.4810 protein using the MAFFT algorithm (Katoh et al. 2009). This alignment identified VAMP-associated proteins (VAPs) as the closest relatives to Tb11.01.4810.

**Immunofluorescence:** Glass slides containing trypanosome cells or trypanosome cytoskeletons (cells treated with 0.1 M PIPES buffer pH 6.9 containing 1% (v/v) nonidet P-40, 2 mM EGTA, 1 mM MgSO_4_, 0.1 mM EDTA) were fixed in 4% paraformaldehyde for 15 minutes and then plunged in -20 °C methanol for 20 minutes; or fixed directly in methanol. Cells were labelled with one or more of the following primary antibodies: BB2 (anti-Ty tag), L6B3 (anti FAZ1) and L8C4 (anti-PFR2). Labelling was visualised with anti-mouse IgG1-Alexa Fluor 488 and/or anti-mouse IgM-Alexa Fluor 594 (Invitrogen). Slides were mounted on DAPI containing Vectashield and examined on a Zeiss Axioplan 2 microscope.

**Generation of transgenic** ***T. brucei*** **cell lines:** Purified linearized plasmid DNA was used to transfected logarithmically growing cultures of procyclic form 29.13 *T. brucei* by electroporation (3 x 100 ms pulses of 1700 V). Stably transfected cells were selected by growth in media containing the appropriate antibiotic and cloned by limiting dilution. A *T. brucei* cell line expressing Ty-TbVAP was generated by inserting the coding sequence of TbVAP immediately downstream of the Ty tag in the pEnT6B vector (Kelly et al. 2007). Transfected cells were selected by growth in medium containing 10 mg.ml^−1^ blasticidin. To generate cells capable of inducible RNAi of TbVAP, the entire TbVAP open reading frame was amplified from *T. brucei* genomic DNA and inserted into the p2T7-177-GTW vector (Lacomble et al. 2009b). Transfected cells were selected by growth in medium containing 5 μg.ml^−1^ of phleomycin.

**Western blotting:** Whole cell extracts of procyclic form *T. brucei* were prepared by re-suspending cell pellets in boiling Laemmli sample buffer and incubating at 95 °C for 5 min. Proteins were resolved in 10% SDS-PAGE gels and transferred to nitrocellulose membranes. These were blocked in 5% milk in TBST (Tris-HCl pH 7.4 containing 0.05% (w/v) Tween20) prior to incubation in 1% milk/TBST containing primary antibodies (BB2 or L8C4, to detect Ty-TbVAP or PFR2, respectively). After 5 washes in TBST, membranes were incubated in 1% milk/TBST containing HRP-conjugated secondary antibodies. Final detection was performed by enhanced chemiluminescence (ECL) according to the manufacturer's instructions (Perkin Elmer, NEL100). A slight modification of this protocol was used for relative quantification of Ty-TbVAP (Supplementary Material Fig. S2). In this case, proteins were transferred to Immobilion-FL PVDF membranes (Millipore) and the secondary antibody used was the GAM-IRDye 800CW (LI-COR). For detection, the Odyssey Infrared Imaging system was used, following manufacturer's instructions. Finally, densitometry analysis of protein bands was performed using the ImageJ software.

**Population growth and cell cycle analyses:** Growth curves were performed as previously described (Vaughan et al. 2008). Cell cycle analysis was performed by counting cells at different stages of the cell cycle in slides of fixed trypanosomes labelled with DAPI as described above. At least 500 cells were counted for each time point.

## Figures and Tables

**Figure:**
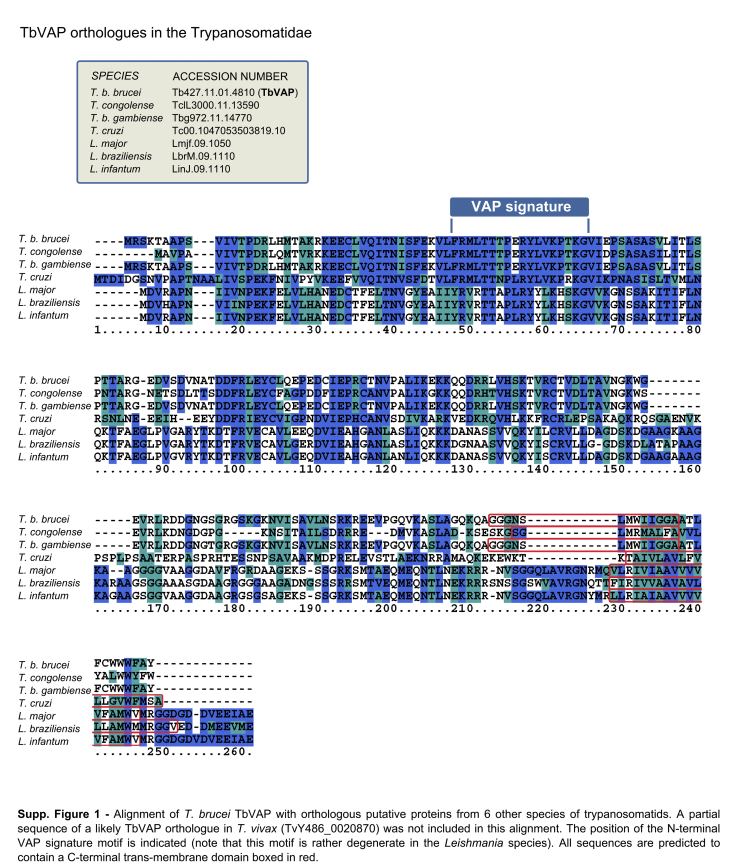


**Figure:**
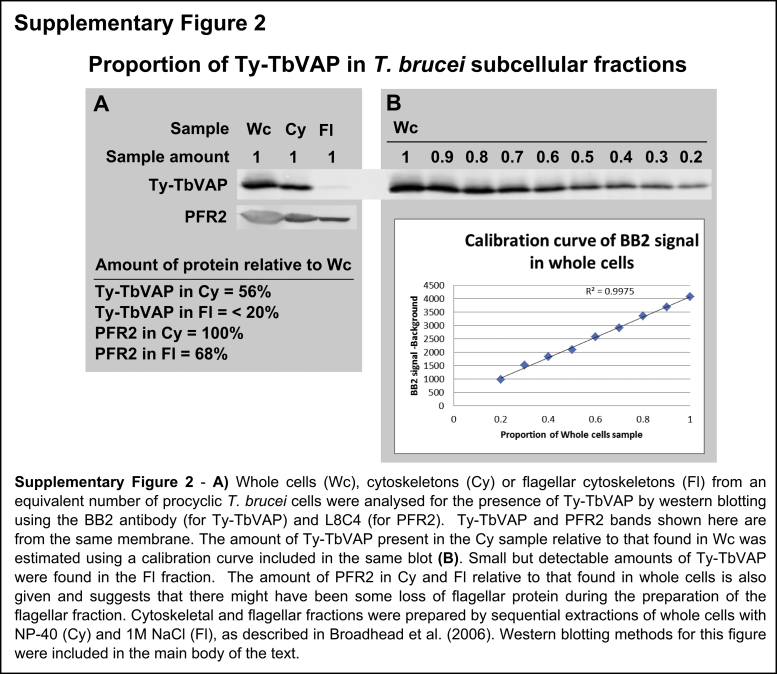


**Figure 1:**
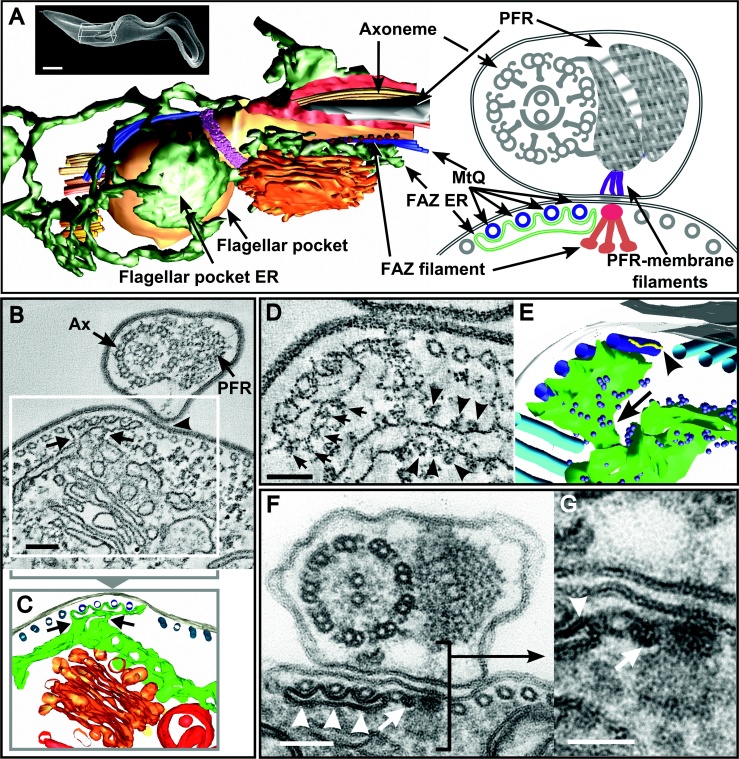
EM tomography analysis of the FAZ ER. **A)** Illustration of the FAZ as viewed in longitudinal 3D models (left, adapted from Lacomble et al., 2009a) or by cross-sections of TEM (right). On the top left of the tomography image, a scanning electron micrograph of a procyclic trypanosome (scale bar, 1 μm) shows the area covered by the 3D model. **B)** Slice of a tomogram through the FAZ area immediately anterior to the flagellar pocket, viewed from the posterior of the cell. Next to the flagellar axoneme (Ax), the paraflagellar rod (PFR) is linked to the membrane at the point of contact between the flagellum and the cell body (arrowhead). In the cytoplasm immediately to the left of this point, the 4 microtubules of the MtQ are interdigitated by the FAZ ER, which is connected to a section of central ER (arrows). Scale bar, 100 nm. **C)** Tomographic reconstruction of the FAZ ER and neighbouring structures in the area boxed in A. Upon reconstruction, the two junctions between the FAZ ER and the central ER appear as ‘tube-like’ membrane bridges (arrows). **D)** Slice of a tomogram in the FAZ area (likely anterior to the nucleus), showing ribosomes associated with the FAZ ER (arrows), indistinguishable from those found in a neighbouring section of rough ER (arrowheads). Scale bar, 50 nm. **E)** 3D reconstruction of the FAZ ER from the area in C, showing associated ribosomes (purple spheres) and a clear tube-like bridge linking it to the rough ER (arrow). In this tomogram, only 3 of the 4 MtQ microtubules appeared encircled by ER membranes, and a “reduced microtubule” (arrowhead) appeared as a thin rod immediately to the right of the MtQ. **F)** TEM cross-section through the FAZ showing 3 MtQ microtubules with associated FAZ ER (arrowheads) and the “reduced microtubule” to the right of the MtQ (arrow). Scale bar, 100 nm. **G)** Larger magnification of a section of E highlighting the membranous nature of the “reduced microtubule” (arrow), which will be referred to henceforth as ‘FAZ tubule‘. Note the similarity between the walls of the FAZ tubule and the membrane of the FAZ ER nearby (arrowhead). Scale bar, 50 nm. **Colour scheme in C and E**: subpellicular microtubules, light blue; MtQ microtubules, dark blue; ER, green; ribosomes, purple; Golgi complex, orange; endosomes, red; plasma membrane, grey; “reduced microtubule” or FAZ tubule, yellow.

**Figure 2:**
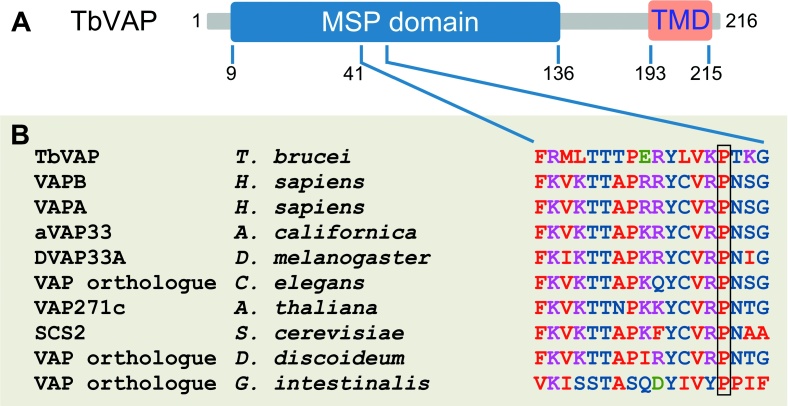
The *T. brucei* VAMP-associated protein orthologue TbVAP. **A)** The domain organisation of TbVAP, with a major sperm protein (MSP) domain at its N-terminal half and a trans-membrane domain (TMD) at the C-terminus. **B)** Alignment of the VAP signature motif of TbVAP and that of 9 other members of the VAP family. The box highlights the proline residue whose mutation to a serine in human VAP-B was linked to three forms of familial motor neuron disease.

**Figure 3:**
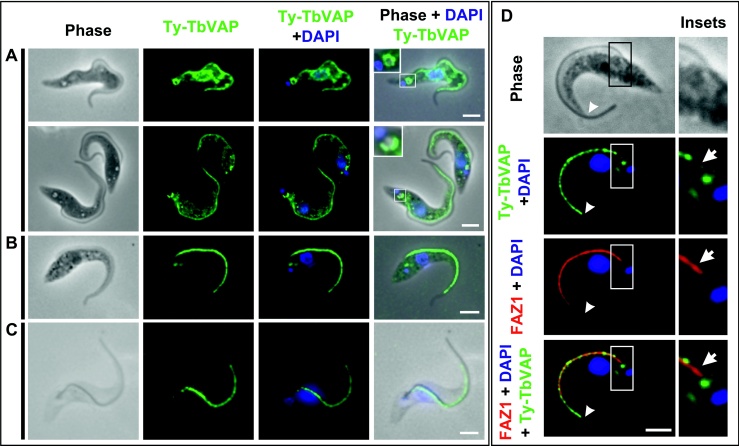
Immunolocalisation of TbVAP in cells expressing Ty-TbVAP. In **A-D**, Ty-TbVAP (recognised by the BB2 antibody) is in green and the nuclear and kinetoplast DNA stained with DAPI are in blue. **A)** In paraformaldehyde fixed cells, BB2 labelling is stronger along the FAZ and at the flagellar pocket (insets). BB2 labelling is also found in discrete structures throughout the cytoplasm. **B)** In methanol fixed cells, Ty-TbVAP can still be detected along the FAZ and in the flagellar pocket area, but labelling throughout the cytoplasm is lost. **C)** In detergent extracted cells, only the FAZ is labelled by BB2. **D**) Methanol fixed cells expressing Ty-TbVAP double labelled with BB2 and L3B2, which recognises the FAZ filament component FAZ1 (in red). Ty-TbVAP co-localises with FAZ1 along the FAZ. However, FAZ1 is present close to flagellar pocket area in a region where TyTbVAP is absent (insets, arrows), while staining for TyTbVAP is stronger than that for FAZ 1 at the anterior of the FAZ (arrowheads). Scale bars, 3 μm.

**Figure 4:**
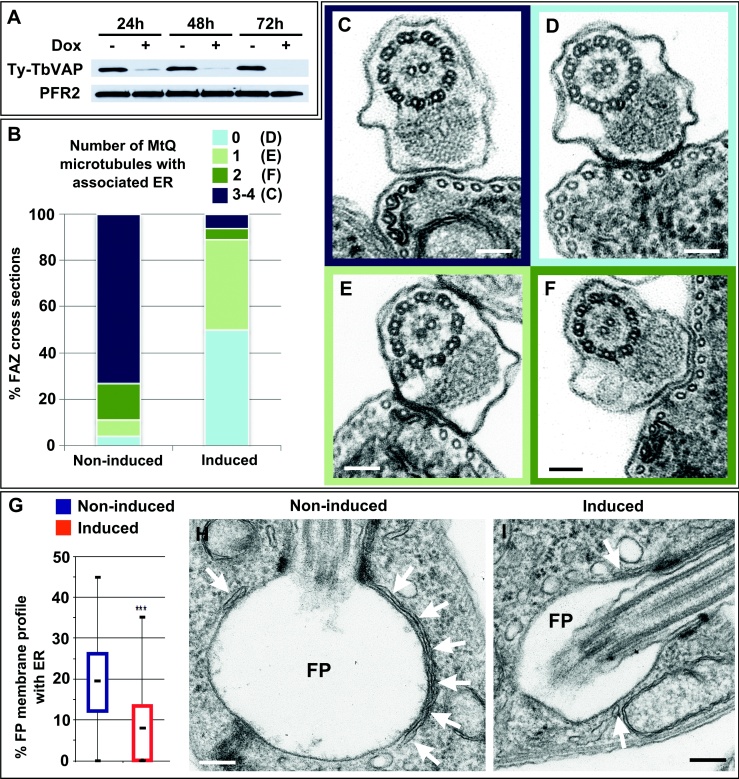
Knockdown of TbVAP by RNAi affects the FAZ and flagellar pocket ER domains. **A)** Western blots of total protein from TbVAP RNAi cells labelled with BB2 (to detect TyTbVAP) or L8C4 (to detect PFR2). As indicated, cells were harvested 24, 48 or 72 h after induction of RNAi by doxycycline (Dox). No Ty-TbVAP could be detected after 72 h of RNAi induction. **B)** Quantitative analysis of the effect of TbVAP RNAi (72 h post-induction) on the FAZ ER, displayed as a percentage of TEM cross-sections through the FAZ (Y axis) showing 0 (light blue), 1 (light green), 2 (dark green) or 3-4 (dark blue) MtQ microtubules with associated FAZ ER. As indicated in brackets, an example image of each of these categories can be found in panels C-F, whose edge is colour coded according to the legend in B. The image in **C** is from a non-induced sample and **D-F** are from doxycycline treated samples (scale bars, 100 nm). **G)** Quantification of the effect of TbVAP RNAi on the association of the ER with the flagellar pocket membrane. The % length of flagellar pocket membrane profiles with associated ER (as measured in TEM sections) was compared between induced and non-induced samples, showing that induced cells have significantly less ER associated with the flagellar pocket (p < 0.01). H) Typical image of the flagellar pocket (FP) of a non-induced cell, showing profiles of ER membrane (arrows) closely apposed to the flagellar pocket membrane. I) Typical image of the flagellar pocket of an induced cell, showing only small sections of the flagellar pocket membrane with associated ER (arrows). Scale bars in H and I, 200 nm.

**Figure 5:**
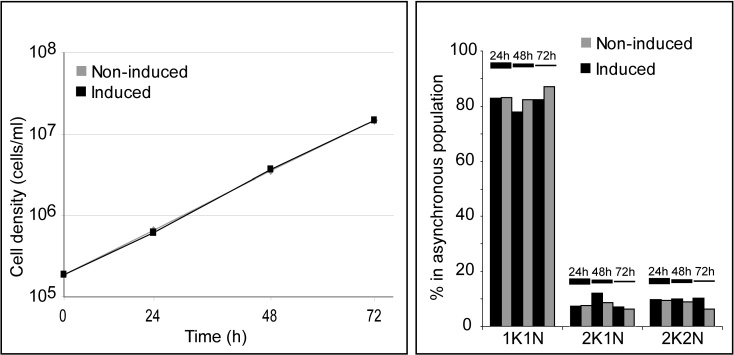
The viability and cell cycle progression of *T. brucei* procyclic cells is not affected by TbVAP RNAi. **A)** Population growth curve of the TbVAP RNAi cells in the presence (black) or absence (grey) of doxycycline in the culture medium. Population growth was followed through a period of 72 h. **B)** Cell cycle analysis of TbVAP RNAi cells in the presence (black) or absence (grey) of doxycycline in the culture medium. The graph displays the % of cells in the classic cell cycle stages (1K1N, 2K1N and 2K2N) 24, 48 and 72 h after RNAi induction.
