# Supplementary material for: A Trypanosoma brucei Protein Required for Maintenance of the Flagellum Attachment Zone and Flagellar Pocket ER Domains
Source: Protist. 2012 Jul;163(4):602–15. doi: 10.1016/j.protis.2011.10.010 (PMC3405529; doi:10.1016/j.protis.2011.10.010)
Supplement: Supplementary file 1 [file mmc1.pdf]

Legends for Movies

Movie 1a – Tomography cross-section showing the flagellum and FAZ of a bloodstream form cell, in the area immediately anterior to the flagellar pocket (identified by the presence of the Golgi complex).

Movie 1b – 3D reconstruction of FAZ components from the section in movie 1a. **Colour scheme**: subpellicular microtubules, light blue; MtQ microtubules, dark blue; ER, green; ribosomes, purple; Golgi complex, orange; endosomes, red; plasma membrane, grey; “reduced microtubule” or FAZ tubule, yellow.

Movie 2a – Tomography cross-section showing the flagellum and FAZ of a bloodstream form cell, likely from the anterior portion of the cell, between the nucleus and the anterior end.

Movie 2b – 3D reconstruction of FAZ components from the section in movie 2a. Colour scheme as in Movie 1b.
