# Supplementary figures and images for: A Trypanosoma brucei Protein Required for Maintenance of the Flagellum Attachment Zone and Flagellar Pocket ER Domains
Source: Protist. 2012 Jul;163(4):602–15. doi: 10.1016/j.protis.2011.10.010 (PMC3405529; doi:10.1016/j.protis.2011.10.010)

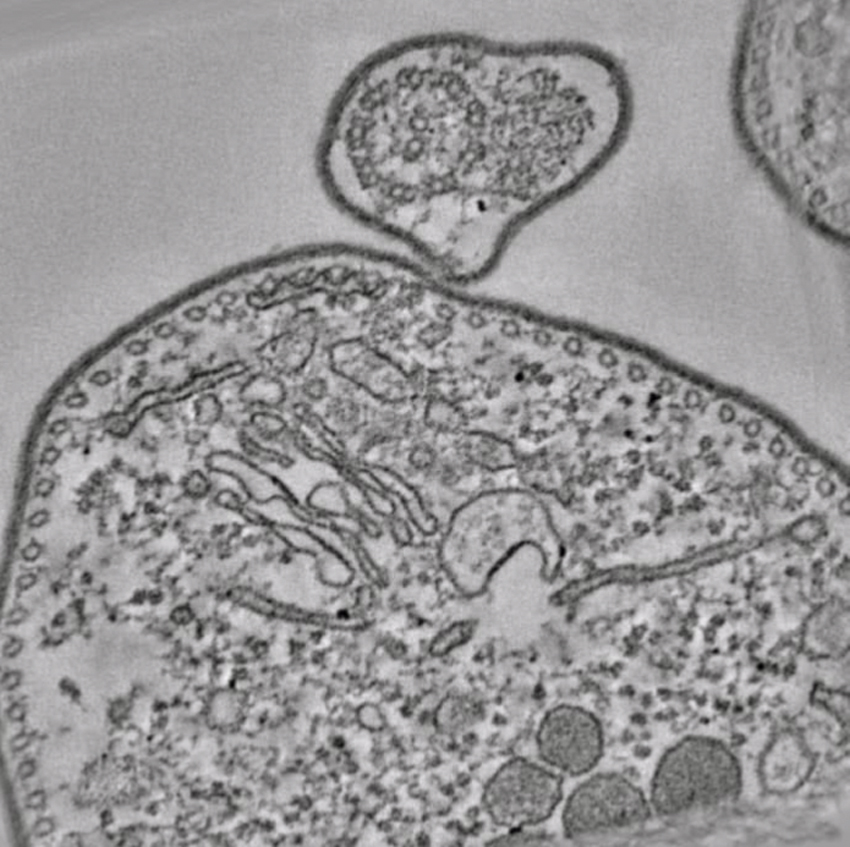

Supplement: Supplementary file 2 [file mmc2.jpg]

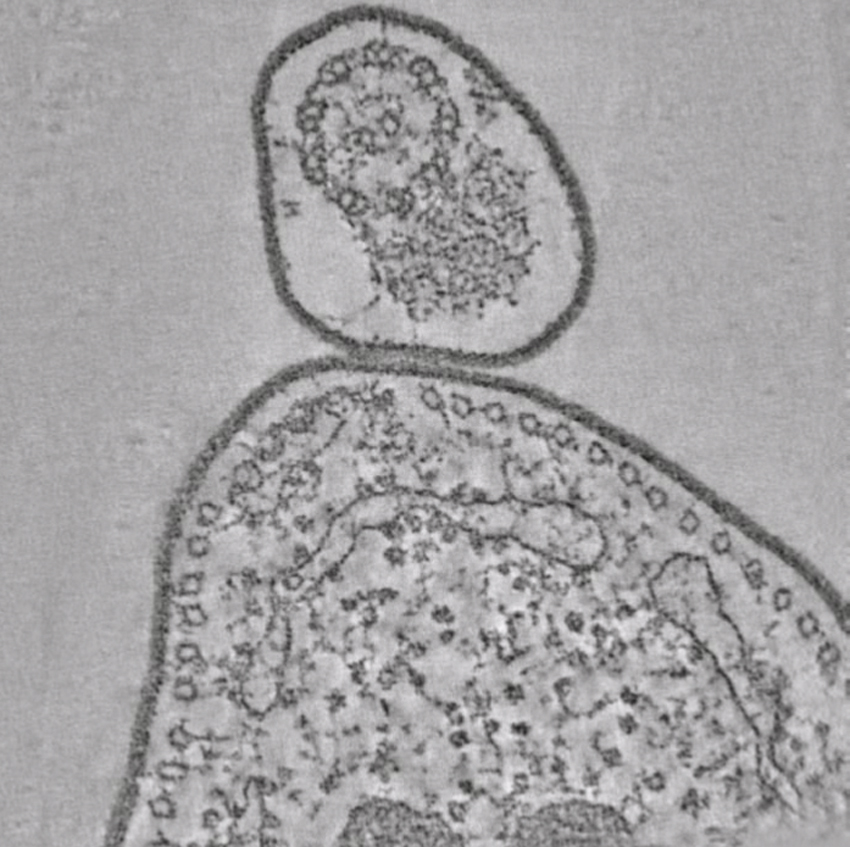

Supplement: Supplementary file 4 [file mmc4.jpg]
